# Modelling the cathodic reduction of 2,4-dichlorophenol in a microbial fuel cell

**DOI:** 10.1007/s00449-022-02699-8

**Published:** 2022-02-09

**Authors:** Luis Fernando Leon-Fernandez, Francisco Jesús Fernandez-Morales, José Villaseñor Camacho

**Affiliations:** Chemical Engineering Department, Institute for Chemical and Environmental Technology ITQUIMA, University of Castilla-La Mancha, Avenida Camilo José Cela S/N 13071, Ciudad Real, Spain

**Keywords:** Mathematical modelling, Microbial fuel cell, Reductive dechlorination, Simulation

## Abstract

This work presents a simplified mathematical model able to predict the performance of a microbial fuel cell (MFC) for the cathodic dechlorination of 2,4-dichlorophenol (2,4-DCP) operating at different cathode pH values (7.0 and 5.0). Experimental data from previous work were utilized for the fitting of the model. The MFC modelled consisted of two chambers (bioanode and abiotic cathode), wherein the catholyte contained 300 mg L^−1^ of 2,4-DCP and the anolyte 1000 mg L^−1^ of sodium acetate. The model considered two mixed microbial populations in the anode compartment using sodium acetate as the carbon source for growth and maintenance: electrogenic and non-electrogenic biomass. 2,4-DCP, its intermediates of the reductive process (2-chlorophenol, 2-CP and 4-chlorophenol, 4-CP) and protons were considered in the model as electron acceptors in the electrogenic mechanism. The global process rate was assumed to be controlled by the biological mechanisms and modelled using multiplicative Monod-type equations. The formulation of a set of differential equations allowed to describe the simultaneous evolution of every component: concentration of sodium acetate in the anodic compartment; and concentration of 2,4-DCP, 2-CP, 4-CP, phenol and chloride in the cathode chamber. Current production and coulombic efficiencies were also estimated from the fitting. It was observed that most of the organic substrate was used by non-electrogenic mechanism. The influence of the Monod parameters was more important than the influence of the biomass yield coefficients. Finally, the model was employed to simulate different scenarios under distinct experimental conditions.

**Supplementary Information:**

The online version contains supplementary material available at 10.1007/s00449-022-02699-8.

## Introduction

Chlorophenols (CPs) are phenol derivatives that contain one or more covalently bonded chlorine atoms. They have been widely used as herbicides, pesticides and disinfectants due to their antimicrobial properties, low biodegradability and environmental persistence. Still, their extended use has led to the contamination of ground and superficial water resources [1, 2]. One representative example of CPs is 2,4-dichlorophenol (2,4-DCP), which has been used in the chemical industry [3], mainly as a raw product for the fabrication of a wide spectrum of pesticides [2], and like most CPs, it presents toxicity and low biodegradability.

Different methods, such as chemical oxidation, advanced oxidation processes, biodegradation, adsorption, ion exchange or liquid–liquid extraction, have been widely studied for the removal of CPs from wastewater, and all of them present advantages but also different limitations to be solved [4]. Reductive dechlorination is one of the treatment options, as CPs accept electrons from an electron donor to break C–Cl bonds because of the electronegative characteristics of chlorine substituents. This process can be achieved by biological, chemical or electrochemical technologies [5–7]. One of the main advantages of CPs dechlorination is that products are less toxic and more biodegradable than the parent molecules [8].

One electrochemical reductive option is the electrocatalytic reductive dichlorination [9], in which protons in an aqueous solution are reduced to atomic hydrogen (H*, a strong reducing agent) on a catalyst-supported cathode (commonly Pd, Pt and Ni), which subsequently attacks and cleaves C−Cl bonds to achieve hydrodechlorination. H* may also evolve into molecular hydrogen (H_2_) as a side reaction at more reductive potentials, competing with the dechlorination pathway [10].

Bioelectrochemical systems (BES) are a recent technology for treatment and resources recovery from wastewaters, and it has been reported that the removal of wastewater pollutants by BESs consumes less energy than conventional electrochemical techniques [1, 11]. BES fundamentals are based on the activity of exoelectrogenic microorganisms which catalyse the electrochemical reactions occurring on the electrode surface of an electrochemical cell. They participate in the electron transfer mechanisms to/from electrode surfaces, which are necessary for the oxidation or reduction of pollutants [12]. BESs can work under different configurations that has led to many different applications. Briefly, a BES system contains electrochemically active microorganisms that oxidize organic matter in the anode compartment and release electrons to the anode surface. Then, electrons are consumed in the cathode compartment to reduce some substances, such as O_2_ to H_2_O, protons to hydrogen gas, and different types of organic pollutants or dissolved metals [13]. Depending on the thermodynamic energy balance, BES can work under microbial fuel cell (MFC) mode as electrical energy can be harvested from the electrical circuit, while under microbial electrolysis cell (MEC) mode electrical energy needs to be supplied to the electrical circuit utilizing a power supply [14].

Organochlorine transformations and removal by means of BES have been studied by different authors. Some works studied the anodic bio-oxidation of CPs [15–18], while other works studied their cathodic dichlorination [19–21]. Coupling bioanodes with the electrocatalytic hydrodechlorination of CPs is recent, and there are only some studies that focus on their feasibility and power consumption compared to conventional pure electrochemical technologies [1, 22].

Substantial efforts have been made for optimizing the electrical requirements/output or yield in BES; however, they involve many processes and parameters, making it challenging. Mathematical models are powerful tools which help to understand the interactions among the parameters, allowing the performance optimization of the BES, i.e. maximizing the electric current output or the conversion of any targeted reactant [23]. Most of the works modelling BESs are recent, based on MFC mode operation. They assume microbial-assisted oxidation on the anode as the limiting stage of the process, disregarding cathodic limitations, i.e. when using open-air cathode for oxygen reduction, considering the latter in excess [23–26]. Some models are based upon a 3D multidimensional description of the anode bioprocess [27, 28], while others draw on time dependence ordinary differential equations for mass balances and transport processes [20, 29]. Most of these proposals describe the microbial kinetics by using Monod or Nernst–Monod derived equations and the electrochemical performance by using Ohm’s law or the Butler–Volmer equation [24].

The present work is based on a previously published paper by the same authors [31], wherein the electrocatalytic hydrodechlorination of 2,4-DCP in a BES working as an MFC under different pH values at the cathode chamber was studied. They found that it was feasible to achieve the transformation of 2,4-DCP by electrocatalytic hydrogenolysis and that greater acidic conditions at the cathode enhanced 2,4-DCP dechlorination and power generation. The present work aims to develop a simplified mathematical model to reproduce the performance of such MFC system for the cathodic removal of 2,4-DCP and to simulate its performance under different operating conditions. The main novelty points of the present work are the following: despite that we propose a simplified grey-box model, compared to some complex models previously reported, it could offer simplicity for design and operation purposes; both phenomena in the anode and cathode compartments are considered since the model includes the influence of all limiting reagents in both compartments; unlike most of the previously reported modelling studies, the final electron acceptor is not oxygen but the chlorinated species and protons; finally, to the author’s knowledge, no previous modelling proposals have been reported when BESs are used for the removal of chlorophenols.

## Materials and methods

### Starting data source: description of the experimental system and operation

The present subsection briefly describes the laboratory-scale BES experimental conditions used by Leon-Fernandez et al. [31] to obtain the experimental data. The MFC setup consisted of two 0.1 L volume chambers (bioanode and abiotic cathode compartments), separated by a proton exchange membrane (PEM, Nafion^®^ 117, DuPont). The anode material was Carbon felt (KFA10, SGL Carbon Group^®^), while carbon cloth (AvCarb^©^ 1071 HCB distributed by Fuel Cell Earth) was used as a cathode, with a Pd loading of 0.5 mg cm^−2^. The MFCs were operated at 25 °C with an external resistor of 120 Ω. The synthetic medium used as catholyte contained 300 mg L^−1^ of 2,4-DCP, using 100 mM phosphate buffer to provide conductivity and to adjust the pH to 7.0 or 5.0, depending on the ratio KH_2_PO_4_–Na_2_HPO_4_. Anolyte pH was 7.44 and it was composed of sodium acetate (1 g L^−1^) and inorganic salts as a growth medium. The seed used for the anodic biofilm development was obtained from the aerobic reactor of a conventional activated sludge facility treating domestic wastewater [32]. MFC systems were operated under reproducible sequencing batch cycles (that is, anolyte and catholyte were replaced after each batch experiment). Experimental data obtained by Leon-Fernandez et al. [31] were discussed in the aforementioned paper, and they are also plotted in the present work (Figs. 2, 3, 4, 5) together with the results of the fittings. Additional details regarding experimental methods are also reported elsewhere [32].

### Conceptual model description

**Fig. 1:**
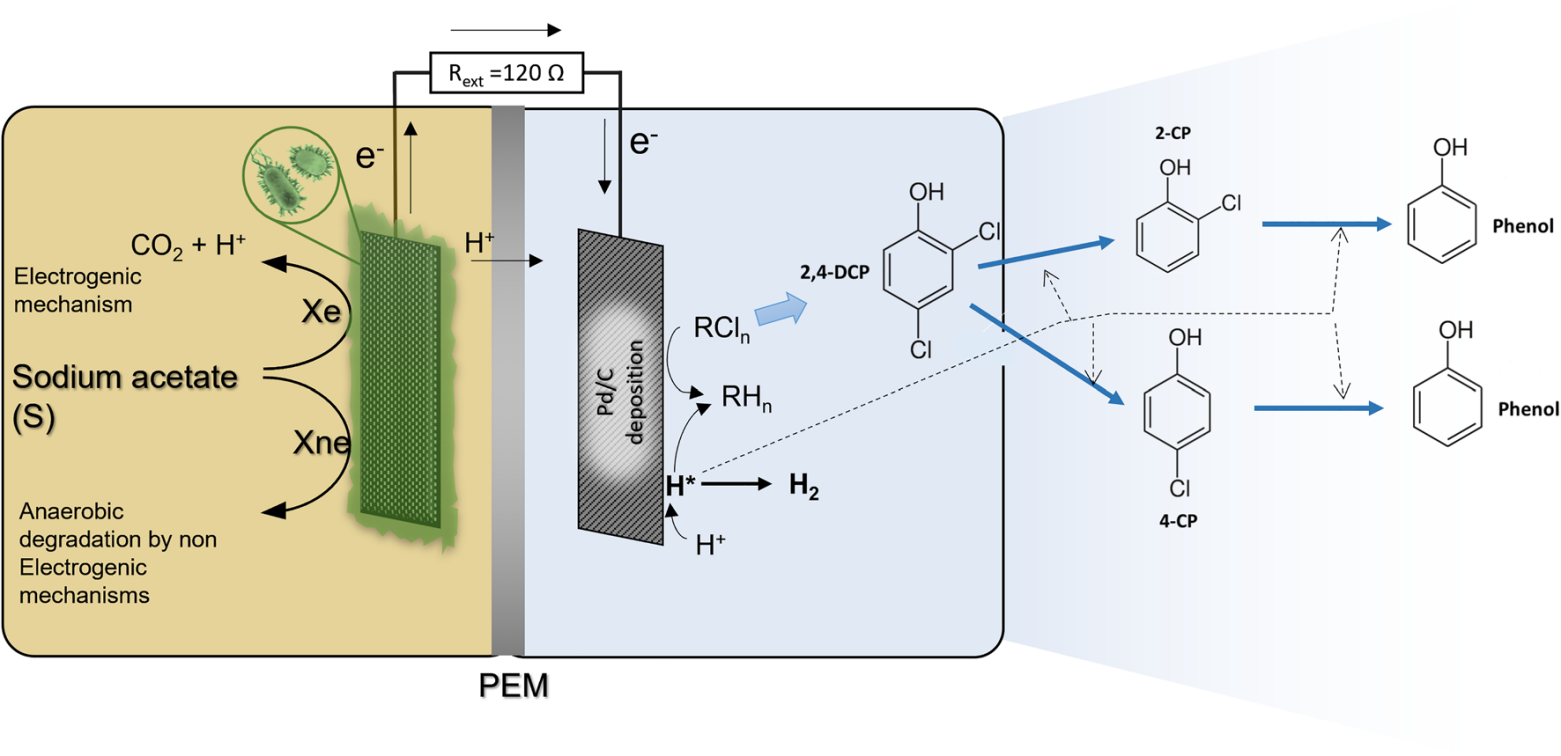
Conceptual description of the processes in the MFC

Figure 1 shows the conceptual description of the MFC for the cathodic 2,4-DCP dechlorination process reported by Leon-Fernandez et al. [31]. Only biological mechanisms were considered for acetate utilization in the anode chamber. The faradaic efficiency values reported [31] (< 50%) showed a decreasing trend due to the growth of non-electroactive species of microorganisms over time, which leads to the biodegradation of the sodium acetate through non-bioelectrochemical pathways. This suggestes that only a low fraction of the acetate was oxidized via exoelectrogenic mechanisms, while the rest could be used under different non-aerobic pathways, such as methanogenesis or denitrification [33, 34]. These non-bioelectrochemical pathways were not studied, as they have been widely studied previously and is not the focus of the present work. Additionally, no acid fermentative processes were considered, as we used directly sodium acetate [34]. Thus, according to this approach, two mixed undefined microbial populations were considered in the present work: electrogenic biomass (X_e_) and non-electrogenic biomass (X_ne_), and each of these two populations use acetate for biomass growth and maintenance.

On the other hand, 2,4-DCP is electrochemically reduced at the cathode to form both 2-CP and 4-CP as intermediates, which are also eventually reduced to phenol. Chloride and phenol concentration increased up to a maximum reached when CPs were removed. According to the electrocatalytic hydrogenolysis mechanism previously described [31], protons (H^+^) in an aqueous solution are reduced to atomic hydrogen (H*) on the cathode surface, which subsequently attacks and cleaves R−Cl bonds of CPs also on the cathode surface to achieve hydrodechlorination [Eq. (1)], where R–Cl is 2,4-DCP or also intermediates 2-CP or 4-CP.

1 $${\text{R}} - {\text{Cl }} + {\text{ 2H}}^* \to {\text{R}} - {\text{H }} + {\text{ HCl}}{.}$$

Additionally, H* may also evolve into molecular hydrogen (H_2_). After depletion of 2,4-DCP, 2-CP and 4-CP, a low electric current and H_2_ production remain since H^+^ reduction to H_2_ continues if there is no limitation in acetate concentration.

According to the reported results [31], anoxic conditions were considered for the model in both compartments as they were closed to the atmosphere and purged with N_2_ prior to the experiments; in addition, CPs’ adsorption on the cathode or membrane surface was neglected.

The model formulation considers the following assumptions to simplify and achieve a fast numerical solution:

- Time-dependent ordinary differential equations are used to describe mass balances. Ideal complete mixing is considered in both anodic and cathodic compartments.
- The viable biomass (both electrogenic and non-electrogenic cultures) is attached to the anode surface while suspended biomass concentration is negligible and continuously washed out.
- Direct electron transfer (without chemical mediators) between cultures and electrodes is considered in the model.
- The model considers a global process rate including simultaneous mechanisms in both anodic and cathodic compartments. The favourable cell voltage allows spontaneity in the reactions taking place in both compartments. The rate was assumed to be controlled by the biological mechanisms where both acetate (electron donor) and final electron acceptors (2,4-DCP, 2-CP, 4-CP and H^+^) concentrations were considered in the multiplicative Monod-type equations. It was observed that the abiotic reference tests did not produce MFC activity or current output, thus biomass activity and concentration would strongly influence the process rate. Because of such a grey-box model proposal, intermediate steps in the electron transfer chain were not considered. A similar model that describes metal removal by BES, also considering processes and mechanisms in both compartments, has been recently reported by the same authors [35].

### Model formulation and parameter estimation

Processes running simultaneously during the batch MFC operation cause changes in the concentration of the following components: X_ne_ (non-electrogenic biomass), X_e_ (electrogenic biomass) and electron donor S (sodium acetate) in the anode chamber; and also concentrations of final electron acceptors (2,4-DCP, 2-CP, 4-CP and H^+^), and concentrations of phenol and chloride in the cathode chamber (H^+^ is also final electron acceptor but its concentration remains constant because of the pH control system used). Also, electricity production is expressed as coulombs transferred from the cathode for the reduction of all final electron acceptors (2,4-DCP, 2-CP, 4-CP and H^+^).

Table 1 shows the abbreviations, meaning, and units of the model parameters, indicating also whether they are estimated through mathematical fitting in the present work or assumed from previously reported works. Table 1 also shows the values of parameters obtained from the model fitting to the MFC performance. The formulation of a system of multiple differential equations would allow to reproduce the simultaneous evolution of every component. Table 2 summarizes the equations system using a Petersen matrix, which has been previously used in similar reported works modelling BES systems [34, 36]. The Petersen matrix includes the processes previously described in the conceptual model description and their corresponding process rates.

**Table 1:** Parameters used in the model

Parameter	Description	Units	Values	Notes and references of previous values reported
Non-electrogenic biological conversion				
${\upmu }_{{{\text{max}},{\text{ne}}}}$	Maximum biomass growth rate	d^−1^	0.51	Estimated [34] (starting iteration value 0.1)
${\text{K}}_{{{\text{S}},{\text{ne}}}}$	Monod half-saturation coefficient for acetate utilization	Mol S L^−1^		None used because of excess acetate concentration
${\text{K}}_{{\text{d}}}$	Endogenous decay rate for active biomass	d^−1^		Assumed as 0.01–0.02 d^−1^ (Zheng et al. [40])
$Y_{{{\text{S}},{\text{ne}}}}$	Biomass yield on acetate for process 1	Mol X_ne_ mol S^−1^	0.024	Estimated [34] (starting iteration value 0.05)
Electrogenic biological conversion				
${\upmu }_{{{\text{max}},{\text{e}}}}$	Maximum biomass growth rate	d^−1^	0.037 (pH 7.0) 0.045 (pH 5.0)	Estimated [38, 39] (starting iteration value 0.1)
${\text{K}}_{{{\text{S}},{\text{e}}}}$	Monod half-saturation coefficient for acetate utilization	Mol S L^−1^		None used because of excess acetate concentration
${\text{K}}_{{24{\text{DCP}}}}$	Monod half-saturation coefficient for 2,4-DCP utilization	Mol 2,4-DCP L^−1^	11.5	Estimated (starting iteration value 5.0)
${\text{K}}_{{2{\text{CP}}}}$	Monod half-saturation coefficient for 2-CP utilization	Mol 2-CP L^−1^	1.6	Estimated (starting iteration value 5.0)
${\text{K}}_{{4{\text{CP}}}}$	Monod half-saturation coefficient for 4-CP utilization	Mol 4-CP L^−1^	4.7	Estimated (starting iteration value 5.0)
${\text{K}}_{{{\text{H}}^{ + } }}$	Monod half-saturation coefficient for H^+^ utilization	Mol H^+^ L^−1^		None used because of constant H^+^ concentration
${\text{K}}_{{\text{d}}}$	Endogenous decay rate for active biomass	d^−1^		Assumed as 0.01–0.02 d^−1^ (Zheng et al. [40])
$Y_{{{\text{S}},24{\text{DCP}}}}$	Biomass yield on acetate for process 3a	Mol X_e_ mol S^−1^	0.096	Estimated [26, 27, 41] (starting iteration value 0.1)
$Y_{{{\text{S}},2{\text{CP}}}}$	Biomass yield on acetate for process 3b	Mol X_e_ mol S^−1^	0.101	Estimated [26, 27, 41] (starting iteration value 0.1)
$Y_{{{\text{S}},4{\text{CP}}}}$	Biomass yield on acetate for process 3c	Mol X_e_ mol S^−1^	0.102	Estimated [26, 27, 41] (starting iteration value 0.1)
$Y_{{{\text{S}},{\text{H}}^{ + } }}$	Biomass yield on acetate for process 3d	Mol X_e_ mol S^−1^	0.100	Estimated (starting iteration value 0.1)
$Y_{{24{\text{DCP}}}}$	Biomass yield on 2,4-DCP	Mol X_e_ mol (2,4-DCP)^−1^	1.7·10^–5^	Estimated (starting iteration value 10^–5^)
$Y_{{2{\text{CP}}}}$	Biomass yield on 2-CP	Mol X_e_ mol (2-CP)^−1^	7.6·10^–5^	Estimated (starting iteration value 10^–5^)
$Y_{{4{\text{CP}}}}$	Biomass yield on 4-CP	Mol X_e_ mol (4-CP)^−1^	0.6·10^–5^	Estimated (starting iteration value 10^–5^)
$Y_{{{\text{H}}^{ + } }}$	Biomass yield on H^+^ to H_2_	Mol X_e_ mol (H^+^)^−1^	1.1·10^–4^	Estimated (starting iteration value 10^–5^)
*f*	Molar fraction of 2,4-DCP reduced to 2-CP	–	0.48 (pH 7.0) 0.63 (pH 5.0)	Estimated (starting iteration value 0.5)
Electrochemical conversion. Stoichiometric electron utilization coefficients for bioelectrochemical reduction of:				
$\delta_{{{\text{e}}^{ - } ,24{\text{DCP}}}}$	2,4-DCP reduction	Coulombs mol (2,4-DCP)^−1^	192970.6	Calculated as *n*·*F* (*n* = 2)
$\delta_{{{\text{e}}^{ - } ,2{\text{CP}}}}$	2-CP reduction	Coulombs mol (2-CP)^−1^	192970.6	Calculated as *n*·*F* (*n* = 2)
$\delta_{{{\text{e}}^{ - } ,4{\text{CP}}}}$	4-CP reduction	Coulombs mol (4-CP)^−1^	192970.6	Calculated as *n*·*F* (*n* = 2)
$\delta_{{{\text{e}}^{ - } ,{\text{H}}^{ + } }}$	H^+^ reduction to H_2_	Coulombs mol (H^+^)^−1^	96485.6	Calculated as *n*·*F* (*n* = 1)

*F* Faraday constant (964853 C mol e^−1^); molar mass of X_e_ and X_ne_: 113 g mol^−1^ (Marcus et al. [36])

**Table 2:** Petersen matrix, containing the stoichiometric values (α) for the main processes taking place in the MFC for CPs removal

Process (*j*)	Components (*i*)										Process rate
	X_ne_	X_e_	S	Electricity	2,4-DCP	2-CP	4-CP	Phenol	Cl^−^	H_2_	
1	1		− 1/Y_S,ne_								$\mu_{{{\text{max}},{\text{ne}}}} \left( {\frac{S}{{K_{{S,{\text{ne}}}} + S}}} \right)X_{{{\text{ne}}}}$
2	− 1										$K_{d} X_{{{\text{ne}}}}$
3a		1	− 1/Y_S,24DCP_	δ_e-,24DCP_/Y_24DCP_	− 1/Y_24DCP_	*f*/Y_24DCP_	(1-*f*)/Y_24DCP_		1/Y_24DCP_		$\mu_{{{\text{max}},e}} \left( {\frac{S}{{K_{S,e} + S}}} \right)\left( {\frac{{H^{ + } }}{{K_{{H^{ + } }} + H^{ + } }}} \right)\left( {\frac{{24{\text{DCP}}}}{{K_{{24{\text{DCP}}}} + 24{\text{DCP}}}}} \right)X_{e}$
3b		1	− 1/Y_S,2CP_	δ_e-,2CP_/Y_2CP_		− 1/Y_2CP_		1/Y_2CP_	1/Y_2CP_		$\mu_{{{\text{max}},e}} \left( {\frac{S}{{K_{S,e} + S}}} \right)\left( {\frac{{H^{ + } }}{{K_{{H^{ + } }} + H^{ + } }}} \right)\left( {\frac{{2{\text{CP}}}}{{K_{{2{\text{CP}}}} + 2{\text{CP}}}}} \right)X_{e}$
3c		1	− 1/Y_S,4CP_	δ_e-,4CP_/Y_4CP_			− 1/Y_4CP_	1/Y_4CP_	1/Y_4CP_		$\mu_{{{\text{max}},e}} \left( {\frac{S}{{K_{S,e} + S}}} \right)\left( {\frac{{H^{ + } }}{{K_{{H^{ + } }} + H^{ + } }}} \right)\left( {\frac{{4{\text{CP}}}}{{K_{{4{\text{CP}}}} + 4{\text{CP}}}}} \right)X_{e}$
3d		1	− 1/Y_S,H+_	δ_e-,H+_/Y_H+_						0.5/Y_H+_	$\mu_{{{\text{max}},e}} \left( {\frac{S}{{K_{S,e} + S}}} \right)\left( {\frac{{H^{ + } }}{{K_{{H^{ + } }} + H^{ + } }}} \right)X_{e}$
4		− 1									$K_{d} X_{e}$

Process (*j*) names: (1) non-electrogenic biomass growth coupled to acetate utilization under anaerobic conditions; (2) non-electrogenic biomass death and endogenous respiration; (3) electrogenic biomass growth coupled to acetate utilization and reduction of 2,4-DCP (3a), 2-CP (3b), 4-CP (3c) and H^+^ to H_2_ (3d); (4) electrogenic biomass death and endogenous respiration

The process rate equations, similar to previously reported works [24, 29, 30], are the following:

Process 1: non-electrogenic biomass growth coupled to acetate utilization under anaerobic conditions.

2 $$r_{1} = r_{{X,{\text{ne}}}} = \mu_{{\text{max,ne}}} \left( {\frac{S}{{K_{{S,{\text{ne}}}} + S}}} \right)X_{{{\text{ne}}}} .$$

Process 2: non-electrogenic biomass death and endogenous respiration.

3 $$r_{2} = r_{{\text{end,ne}}} = K_{d} X_{{{\text{ne}}}} .$$

Process 3: electrogenic biomass growth coupled to acetate utilization at the bioanode, extracellular electron transfer and reduction of the following four final electron acceptors at the cathode surface: 2,4-DCP (process 3a), 2-CP (process 3b), 4-CP (process 3c) and H^+^ to H_2_ (process 3d).

4 $$r_{3a} = r_{{X,{\text{24DCP}}}} = \mu_{{{\text{max}},e}} \left( {\frac{S}{{K_{S,e} + S}}} \right)\left( {\frac{{H^{ + } }}{{K_{{H^{ + } }} + H^{ + } }}} \right)\left( {\frac{{24{\text{DCP}}}}{{K_{{{\text{24DCP}}}} + 24{\text{DCP}}}}} \right)X_{e} ,$$

5 $$r_{3b} = r_{{X,{\text{2CP}}}} = \mu_{{\text{max,e}}} \left( {\frac{S}{{K_{S,e} + S}}} \right)\left( {\frac{{H^{ + } }}{{K_{{H^{ + } }} + H^{ + } }}} \right)\left( {\frac{{2{\text{CP}}}}{{K_{{2{\text{CP}}}} + 2{\text{CP}}}}} \right)X_{e} ,$$

6 $$r_{3c} = r_{{X,4{\text{CP}}}} = \mu_{{{\text{max}},e}} \left( {\frac{S}{{K_{S,e} + S}}} \right)\left( {\frac{{H^{ + } }}{{K_{{H^{ + } }} + H^{ + } }}} \right)\left( {\frac{{4{\text{CP}}}}{{K_{{4{\text{CP}}}} + 4{\text{CP}}}}} \right)X_{e} ,$$

7 $$r_{3d} = r_{{X,H^{ + } }} = \mu_{{{\text{max}},e}} \left( {\frac{S}{{K_{S,e} + S}}} \right)\left( {\frac{{H^{ + } }}{{K_{{H^{ + } }} + H^{ + } }}} \right)X_{e} .$$

Process 4: electrogenic biomass death and endogenous respiration.

8 $$r_{4} = r_{{{\text{end}},e}} = K_{d} X_{e} .$$

As a result, the differential mass balance that describes the time-dependent evolution of the concentration of each component (C_i_) will be:

9 $$\frac{{{\text{d}}C_{i} }}{{{\text{d}}t}} = \mathop \sum \limits_{j} \alpha_{j} \times r_{j} ,$$

where α is the stoichiometric value (Table 2) corresponding to component *i* in process *j*, and *r*_*j*_ is the process reaction rate [Eqs. (2) to (8)]. A system of ten differential equations is obtained to describe the evolution of concentrations of X_e_, X_ne_, S, 2,4-DCP, 2-CP, 4-CP, phenol and chloride, accumulated H_2_ generation (mmol) and electricity generation (coulombs L^−1^ d^−1^). Stoichiometry of processes 3a, 3b and 3c are indicated in Eq. (1). However, stoichiometry of 2,4-DCP dechlorination is not known, as it can be reduced both to 2-CP or 4-CP, and *f* has been defined as a parameter that indicates the molar 2,4-DCP fraction that is reduced to 2-CP according to Eq. (10). Finally, the stoichiometry of H_2_ generation is H* 0.5 H_2_.

10 $${2},{4} -  {\text{DCP }} + {\text{ 2H}}^{*} \to f \, {2} - {\text{CP }} + \, \left( {{1} - f} \right)\,{ 4} - {\text{CP }} + {\text{ HCl}}{.}$$

The differential mass balance equations were solved simultaneously by implementing the Gauss–Newton algorithm as reported in the literature [34], fitting them to the experimental data set. For the fitting of the model, the “solver” tool (GRG Nonlinear method) in MS Excel was utilized. An initial set of values were assigned to the parameters to be estimated and after several iterations, the values of the parameters that yielded the lowest sum of squared error (SSE) were selected. The minimum SSE value was determined from Eq. (11), where *n* is the number of data points, and C_i,exp_ and C_i,model_ are the experimental values and the model prediction values, respectively, of the concentrations of every component considered in the model description.

11 $${\text{SSE = }}\mathop \sum \limits_{i = 1}^{n} \left( {\left( {C_{{i,{\text{exp}}}} - C_{{i,{\text{model}}}} } \right)_{i}^{2} } \right).$$

A correct set of initial values is necessary to start the mathematical iteration process and obtain an accurate estimation of the model’s parameters [37]. These initial values guarantee the convergence of the mathematical functions to obtain a local minimal SSE and thus it is important to select them correctly. Mathematical fitting was performed according to two criteria: (1) the values must present biological sense and they must be included within the usual ranges and (2) they must approximately reproduce experimental data. Our priority was not to obtain the maximum accuracy and minimum differences between data and model predictions, but that the whole set of estimated kinetic and stoichiometric values would present correct physical sense.

The following simplifications or assumptions were used.

- It was assumed that sodium acetate concentration was always high enough to consider that electron donor concentration was not rate limiting and thus it was not considered in the Monod equation; therefore, the corresponding Monod half-saturation constants (K_S,e_ and K_S,ne_) were not used. Additionally, the concentration of organic matter from decayed biomass would be negligible compared to the no limiting acetate concentration.
- In the same way, since H^+^ concentration in the catholyte remained constant due to the phosphate buffer, the corresponding Monod multiplicative term in Eqs. (4) to (7) was constant and it was included in the µ_max,e_ value; then µ_max,e_ is an apparent constant that depends on the catholyte pH and K_H+_ was not fitted. Initial maximum biomass growth rate values (µ_max,ne_ and µ_max,e_) were selected according to previously reported typical values of biomass growth rate for non-bioelectrogenic heterotrophic anaerobic biomass [34] and bioelectrogenic biomass [38, 39].
- The cathode polarization due to the modification of the external resistance or to external voltage supply would also influence the exerted current, typically modelled with Butler–Volmer type kinetics (exponential factor containing the electrode overpotential). As not only the bioprocess but also the cathode reactions have many processes involved with no straightforward kinetics (i.e., several parallel reactions on the cathode), the factor containing the electrode polarization is also included within the µ_max,e_ parameter (similarly to the previous point). If the external resistor was modified, a new fitting of µ_max,e_ parameter would be required.
- The initial values of biofilm biomass concentration in experiments were very low [31] and the initial concentration of the active biomass used for calculations was 5.0 and 1.0 mg L^−1^ for X_e_ and X_ne_, respectively.
- As for the biomass yield coefficients (Y_S,ne_, Y_S,24DCP_, Y_S,2CP_, Y_S,4CP_, Y_S,H+_), it must be noted that biomass growth measurements are not available, which implies a limitation in fitting calculations. The corresponding initial values for estimation of these coefficients were selected again according to previously reported values of biomass yield for non-bioelectrogenic heterotrophic anaerobic biomass [34] and bioelectrogenic biomass [38, 39]. The initial values to estimate Y_24DCP_, Y_2CP_ and Y_4CP_ coefficients were calculated considering the respective previous initial values of Y_S,24DCP_, Y_S,2CP_ and Y_S,4CP_ and the experimental ratio observed in the consumption rates of the organic substrate S and CPs. The sensitivity of these coefficients in the model and the biomass concentration predictions will be discussed in “Results and discussion”. To reduce the number of parameters to be estimated, it was assumed that Y_S,24DCP_, Y_S,2CP_ and Y_S,4CP_ were very similar, as well as Y_24DCP_, Y_2CP_ and Y_4CP_.
- Finally, the endogenous decay rate for active biomass (K_d_) was not estimated and the value reported by Zeng et al. [40] was used (Table 1).

Two different sets of experimental data have been used for model fitting: data from MFC working under two values of catholyte pH (7.0 and 5.0). However, it was considered that the model should adequately reproduce the system performance working under different pH values, but with a unique set of final parameters, except *f* and the apparent value of µ_max,e_ (that is, the influence of catholyte pH would be observed from the variations in *f* and µ_max,e_).

## Results and discussion

**Fig. 2:**
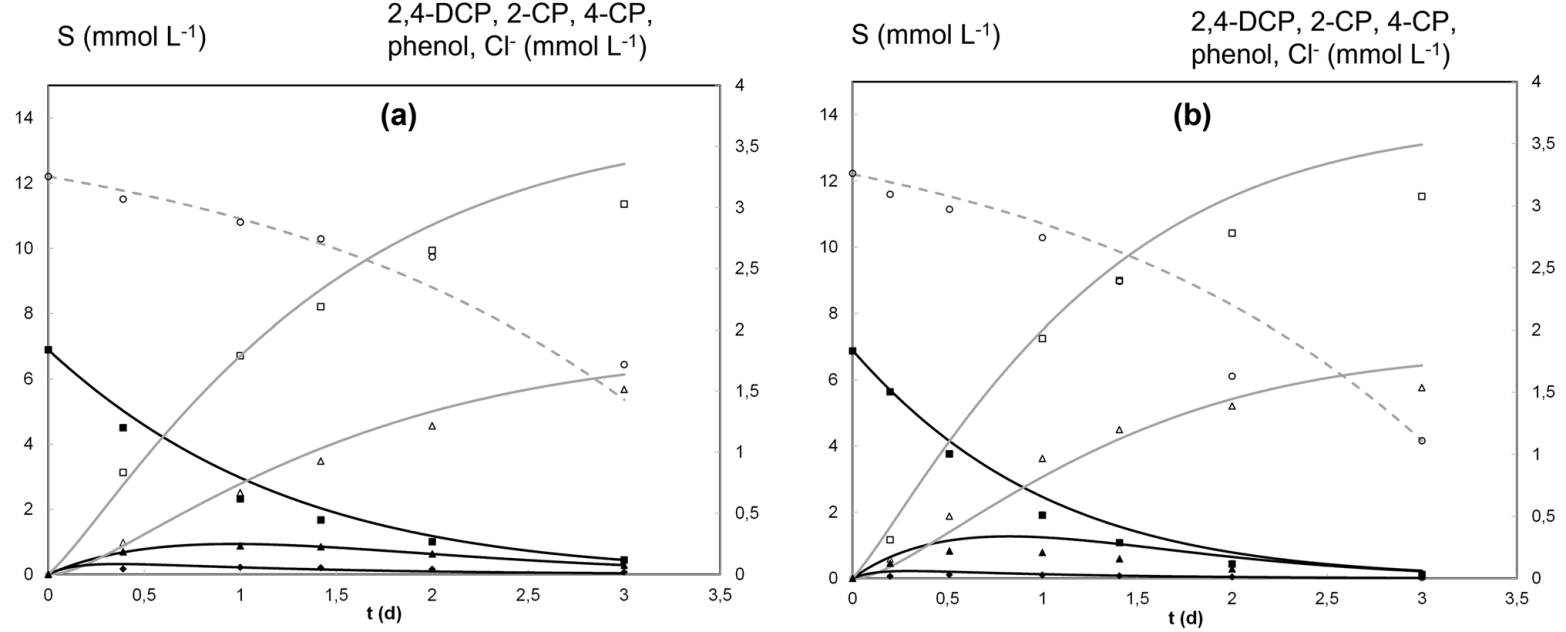
Concentration profiles of S (open circles), 2,4-DCP (filled squares), 2-CP (filled triangles), 4-CP (filled circles), phenol (open triangles) and chloride (open squares) during a batch MFC operation cycle at pH 7.0 (a) and pH 5.0 (b). Experimental values (data points) and model prediction (lines)

**Fig. 3:**
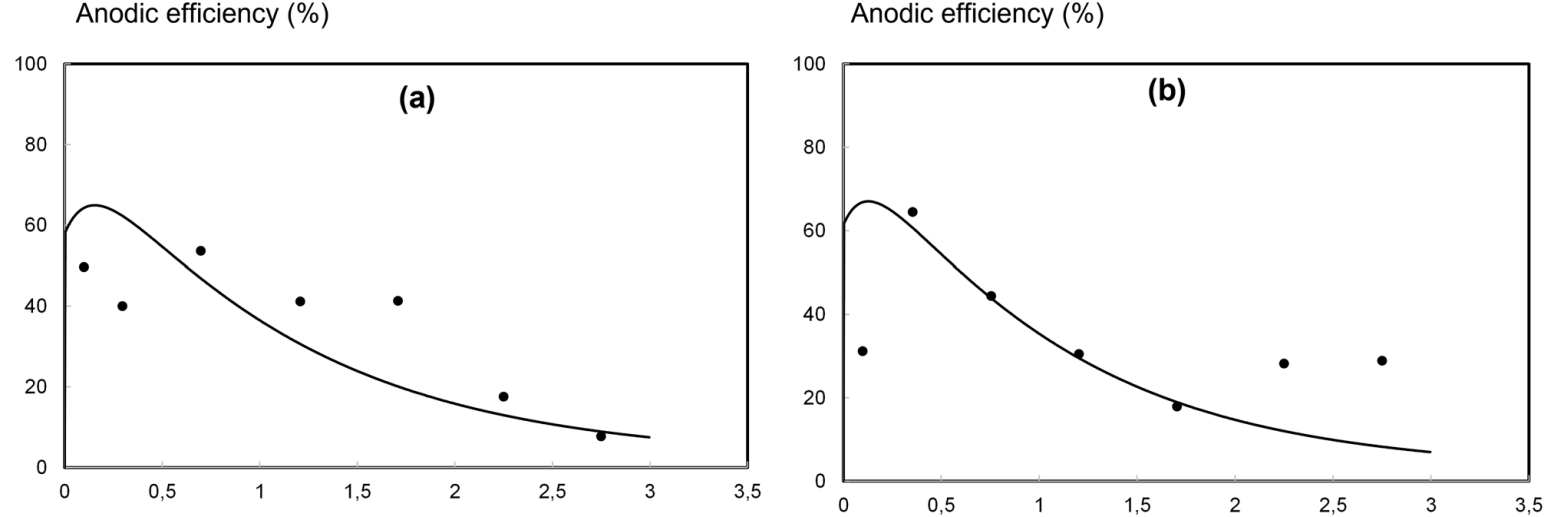
Anodic coulombic efficiency at pH 7.0 (**a**) and pH 5.0 (**b**). Experimental values (data points) and model prediction (lines)

**Fig. 4:**
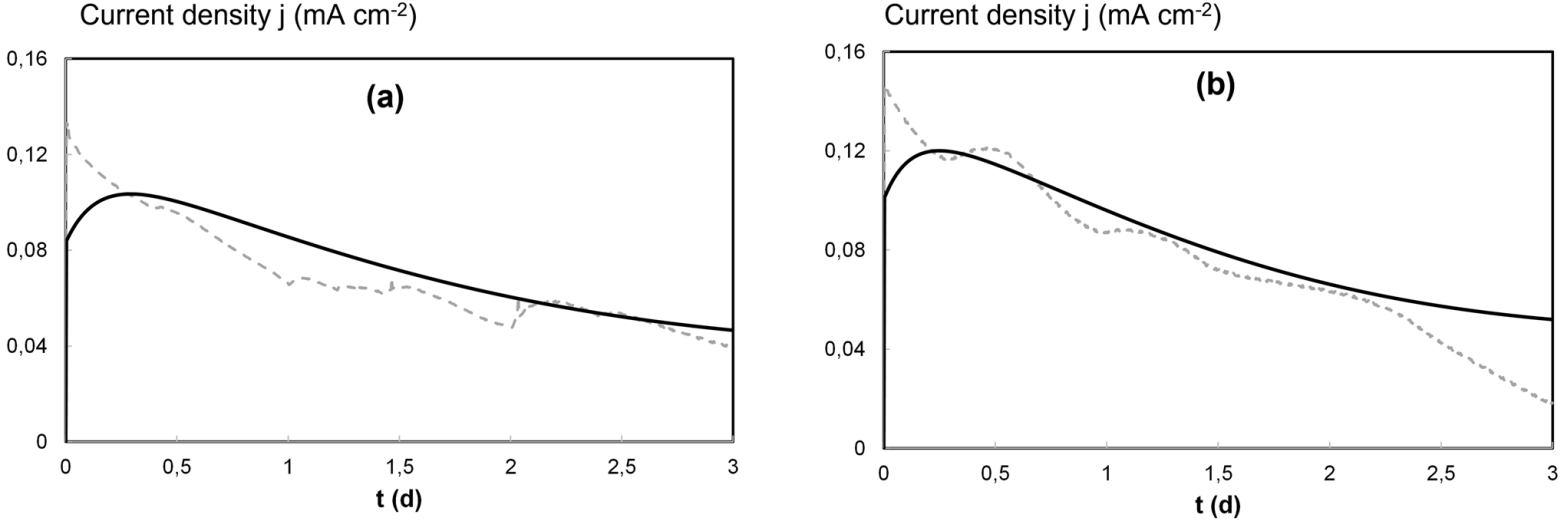
Current density generated in the MFC at pH 7.0 (**a**) and pH 5.0 (**b**). Experimental values (dashed lines) and model prediction (solid lines)

**Fig. 5:**
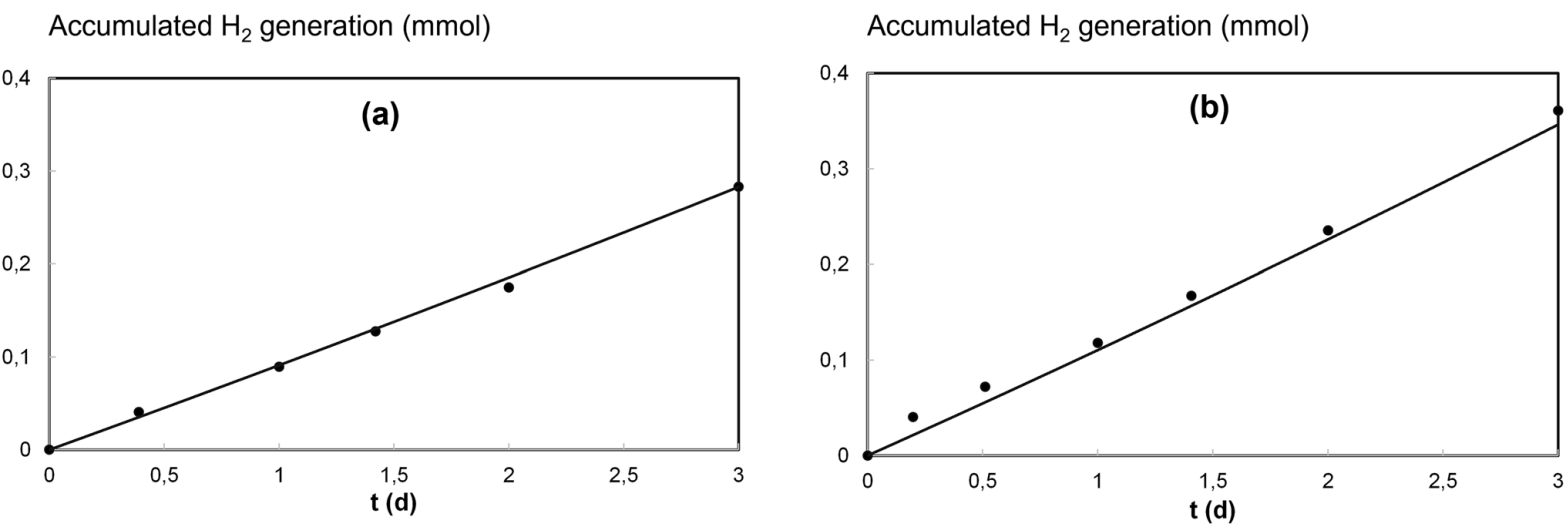
Accumulated H_2_ generation during the experiments. Experimental values (data points) and model prediction (lines)

Figures 2, 3, 4, 5 show the modelling results of MFC working under two different pH values at the catholyte.

Figure 2 shows the experimental results (data points) reported by Leon-Fernandez et al. [31] and the model predictions (lines) regarding the concentrations of S (in anode chamber) and 2,4-DCP, 2-CP, 4-CP, phenol and chloride (in cathode chamber) during a batch MFC operation cycle. Values of the *r*^2^ coefficient for each series are reported in the supplementary material, table SM1. Figure 3 shows the anodic coulombic efficiency: experimental values (data points) and the model predictions (lines). Figure 4 shows the current density profiles during the batch cycles: experimental results (dashed lines) and the model predictions (solid lines). The fitting of experimental results to the mathematical model previously described provided the parameters shown in Table 1.

According to the results shown in Fig. 2, it can be considered that the model adequately describes the time evolution of the different species involved in the anode and cathode chamber phenomena. The most significant deviation between data and theoretical curves corresponds to chloride evolution at the end of the batch experiments. As observed, sodium acetate concentration after 3 h can be considered high enough (6 and 4 mmol L^−1^ approximately at the end of experiments at pH 7.0 and 5.0, respectively) to assume that it is not rate limiting. The low values of the Monod half-saturation coefficients K_2CP_ and K_4CP_ compared to K_24DCP_ indicate that both intermediates are easily reduced compared to 2,4-DCP and their maximum concentrations, reached after approximately 1 d, are very low. It is observed that a lower pH value increases *f* (Table 1) and then favours the formation of 2-CP vs 4-CP. It was observed that the model parameters offered different sensitivity levels regarding the fitting between data points and the model. Supplementary Figure S1 shows the results of model predictions under changes of ±15% in the values of the estimated parameters (Table 1). It can be observed that the influence of the Monod parameters (µ_max,e_ values as well as the half-saturation coefficients for electrogenic processes) was significant (Figure S1 a and b), while the influence of the biomass yield coefficients was negligible. Finally, the effect of the endogenous respiration process was also observed to be negligible on the modelling results.

Figure 3 shows that anodic coulombic efficiencies values reached more than 50% at the beginning in both experiments, but then quickly decreased. Considering these values of anodic efficiencies and that µ_max,ne_ value obtained is clearly higher than µ_max,e_ values (Table 1), it can be assumed that most of the organic substrate was used by non-electrogenic mechanisms. Experimental and theoretical coulombic efficiencies were calculated as reported by Fernandez et al. [34] using the experimental data (reagent concentrations and current density) or theoretical model predictions, respectively. Calculations considered not only the stoichiometric electron generation from acetate oxidation (9413.2 C gS^−1^), but also the stoichiometric electron generation from endogenous respiration using decayed biomass (17077.9 C gX_e_^−1^ decayed) as reported by Marcus et al. [36]. Higher deviations between model and data points can be observed compared to Fig. 2. However, the model consistently reproduces the system behaviour and it can be observed that slightly higher efficiency values are obtained under pH 5.0 compared to pH 7.0, as reported [31]. If the faradaic efficiencies of the system is to be promoted, anything that stimulates the electrochemical response (electrical output) would favour the growth of the electroactive culture, increasing the selectivity of oxidizing the organic substrate to CO_2_ and releasing electrons to the electrode. As detailed above, a lower cathode pH promotes cathode reactions that indirectly favour the overall performance, achieving higher faradaic efficiencies; however, there are more options that could be considered. μ_max,ne_ parameter is one order of magnitude higher than μ_max,e_ (see Table 1). The longer the retention time, the lower would be the faradaic efficiencies attained, as the non-electrochemical population grows faster. Therefore, shorter retention times would favour the faradaic efficiency, although it would limit the total dechlorination achieved. Other possibilities would be to use pure eletrogenic strains (i.e. geobacter), decreasing the external resistance of the cell or even by suppling external voltage input. All of these options would increase the value of μ_max,e_, achieving higher current densities.

It is considered that the values obtained for the biomass yield coefficients (Y_S,ne_, Y_S,24DCP_, Y_S,2CP_, Y_S,4CP_ and Y_S,H+_) can be comparable to other ones reported in previous works [26, 27, 41] although they correspond to different biological substrates (approximate range between 0.2 and 0.05 mol X_e_ mol S^−1^). As previously indicated, no experimental data are available for X_e_ and X_ne_ during the experiments. Absence of experimental values of active biomass concentration has been observed in previous modelling published works, because it presents a technical challenge. Complicated tasks would be necessary such as biofilm separation from electrode porous material and separation and quantification of the concentration of different microbial groups. Picioreanu et al. [42] suggested very low biomass growth and thus approximately constant biomass concentration during an MFC operation. It should be noted that biomass yield values of both anaerobic and bioelectrogenic cultures are quite lower than conventional aerobic biomass yield values.

According to the model developed in the present work, there is a consumption of approximately 8 mmol L^−1^ of sodium acetate after 3 d, but the increase in X_e_ biomass concentration is less than 0.16 mmol L^−1^ and X_ne_ variation is negligible.

Figure 4 reports the current density (*j*, mA cm^−2^) measurements and model predictions. As the current was expressed in C L^−1^ d^−1^, current density was then calculated considering the cathode surface and the corresponding change in time units. The model predicts approximately the low values of electric current production during a batch cycle. Relatively high current generation (between 0.08 and 0.12 mA cm^−2^) at the beginning can be observed, decreasing subsequently. Deviations between data and model are observed during the first 6 h and it could be caused by the interferences of electron acceptors such as low amounts of dissolved oxygen at the beginning of experiments. CPs are nearly depleted after approximately 2.5 d; however, low current values remain. This behavior might correspond to the current caused by process at 3 d (H^+^ reduction to H_2_, once CPs are removed). The model then predicts a final asymptotic *j* value. This behavior agrees with the experimental data in Fig. 4a, but it is not the case in Fig. 4b where deviations between data and model are observed after 2.5 d.

Figure 5 reports accumulated H_2_ generation during the experiments. Data points correspond to H_2_ production calculated from the current efficiency (it was assumed that the difference between the total electron flow from the cathode and the electron utilization for CPs reduction was used for H_2_ generation). Model predictions (lines) accurately reproduce the accumulated H_2_ generation. The linear behavior corresponds to the constant electron acceptor concentration (H^+^) because of pH control in the catholyte. H_2_ generation under pH 5.0 is slightly promoted than at pH 7.0, which agrees with the final *j* values predicted in Fig. 4.

**Fig. 6:**
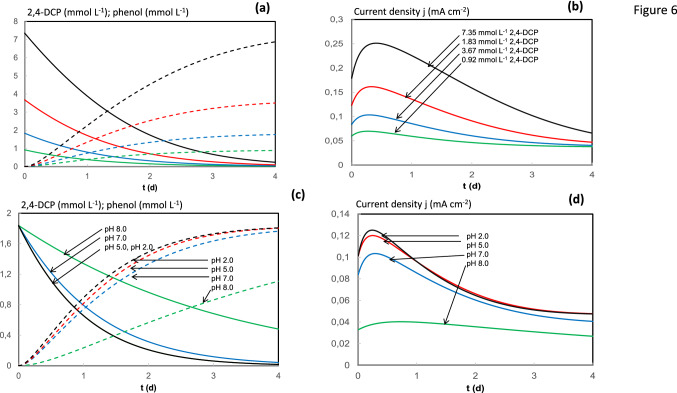
**a,** **b** Simulation results using different 2,4-DCP initial concentrations (black: 0.92 mmol L^−1^; red:1.83 mmol L^−1^, blue: 3.67 mmol L^−1^ and green: 7.35 mmol L^−1^); **c,** **d** simulation results using different catholyte pH values (black: 2.0; red: 5.0, blue: 7.0 and green: 8.0)

Compared to previously published modelling works regarding BES technology, the present work offers a grey-box model which does not provide a deep insight into the phenomena occurring in the BES and, consequently, its application is restricted to concrete situations. However, it is easy to implement and could be used as a practical tool for quick performance of a BES applied for the cathodic treatment of polluted streams, i.e. industrial CPs-polluted waters. In addition, simulations considering changes in experimental conditions can be easily made to predict its performance and give information about expected faradaic efficiencies, removal rates and retention times needed for CPs removal. As examples, Fig. 6 includes simulations of the MFC performance using the developed model. Figures 6a, b shows the predicted concentration profiles of 2,4-DCP (decreasing lines) and phenol (increasing lines) as well as current generation in four simulations using different 2,4-DCP initial concentrations (0.92, 1.83, 3.67 and 7.35 mmol L^−1^). Excess concentration of sodium acetate (24.0 mmol L^−1^) was used in all cases to ensure no fuel limitations at the anode, and the catholyte pH was 7.0. A final asymptotic *j* value can be observed as previously discussed. Figure 6c, d shows the results of four simulations under different catholyte pH values (2.0, 5.0, 7.0 and 8.0): Figure 6c displays the predicted concentration profiles of 2,4-DCP (decreasing lines) and phenol (increasing lines), while Fig. 6d shows the current generation profiles. Calculations in Fig. 6c, d were possible by solving Eq. (12).

12 $$\mu_{{{\text{max}},e}} \left( {\text{apparent value}} \right) = \mu_{{{\text{max}},e}} \left( {\frac{{H^{ + } }}{{K_{{H^{ + } }} + H^{ + } }}} \right).$$

According to the model assumptions, µ_max,e_ apparent values were obtained from fitting calculations depending on the catholyte pH. By solving Eq. (12) at different pH values, real µ_max,e_ as well as K_H_^+^ values can be obtained and simulations profiles are plotted in Fig. 6c, d. The positive influence of lower cathode pH can be observed, as suggested by Leon-Fernandez et al. [31]. Siumulations operating at cathode pH 2.0 and 5.0 are nearly identical. The final asymptotic *j* value increases at lower pH.

Despite the possible limitations of the proposed model, the organic source and its concentration in the anolyte may not be so restrictive. For instance, using low-cost organic substrates, i.e. agrofood wastewater with high content of easily biodegradable organics, would be implementable in the model in terms of chemical oxygen demand concentration. The model parameters could be fitted even by considering the anodic substrate as a limiting factor, a case in which its multiplicative term in the Monod-type kinetics must be included. Some aspects to expand and improve the applicability of this modelling tool could be impl;emented, such as using limiting concentrations of acetate or more complex and low-cost organic substrates. MFC design optimization and control could be facilitated by process modelling, allowing for its deeper understanding and helping to identify its main bottlenecks.

## Conclusions

The grey-box model proposed in this work allowed the estimation of every component concentration profile, while the kinetic and stoichiometric parameters offered coherent values with regard to these types of anaerobic and bioelectrochemical processes. The theoretical current density evolution was simultaneously coupled to the electrochemical reactions on both anode and cathode, and the model results were in agreement with the experimental data. The current simulation tool should be tuned for every specific situation; however, it is easy to implement and could be used to rapidly check the performance of the BESs applied to different cathodic processes, i.e. the electrocatalytic dechlorination of CPs. In this sense, simulations considering changes in experimental conditions can be easily made to predict efficiencies, rates and retention times needed for an optimal reactor design.

## Supplementary Information

Below is the link to the electronic supplementary material.

Supplementary file1 (DOC 28 KB)

Supplementary file2 (TIF 1002 KB) Simulation results under variations of ±15% in the values of the estimated parameters: (a) changes in µmax,e value; (b) changes in Ks values in electrogenic processes; (c) changes in Ys values in electrogenic processes
