# Supplementary material for: Modelling the cathodic reduction of 2,4-dichlorophenol in a microbial fuel cell
Source: Bioprocess Biosyst Eng. 2022 Feb 9;45(4):771–82. doi: 10.1007/s00449-022-02699-8 (PMC8948123; doi:10.1007/s00449-022-02699-8)
Supplement: Supplementary file 1 — Supplementary file1 (DOC 28 KB) [file 449_2022_2699_MOESM1_ESM.doc]

Table SM1. Values of the r^2^ coefficient for each fitting series

|  | **Acetate** | **Current** | **2,4-DCP** | **2-CP** | **4-CP** | **Phenol** | **Cl^-^** | **H_2_** |
| --- | --- | --- | --- | --- | --- | --- | --- | --- |
| **pH 7** | 0.9849 | 0.7950 | 0.9882 | 0.9960 | 0.5545 | 0.9985 | 0.9956 | 0.9977 |
| **pH 5** | 0.9406 | 0.9285 | 0.9951 | 0.9491 | 0.6320 | 0.9772 | 0.9961 | 0.9975 |
