# Supplementary figures and images for: Modelling the cathodic reduction of 2,4-dichlorophenol in a microbial fuel cell
Source: Bioprocess Biosyst Eng. 2022 Feb 9;45(4):771–82. doi: 10.1007/s00449-022-02699-8 (PMC8948123; doi:10.1007/s00449-022-02699-8)

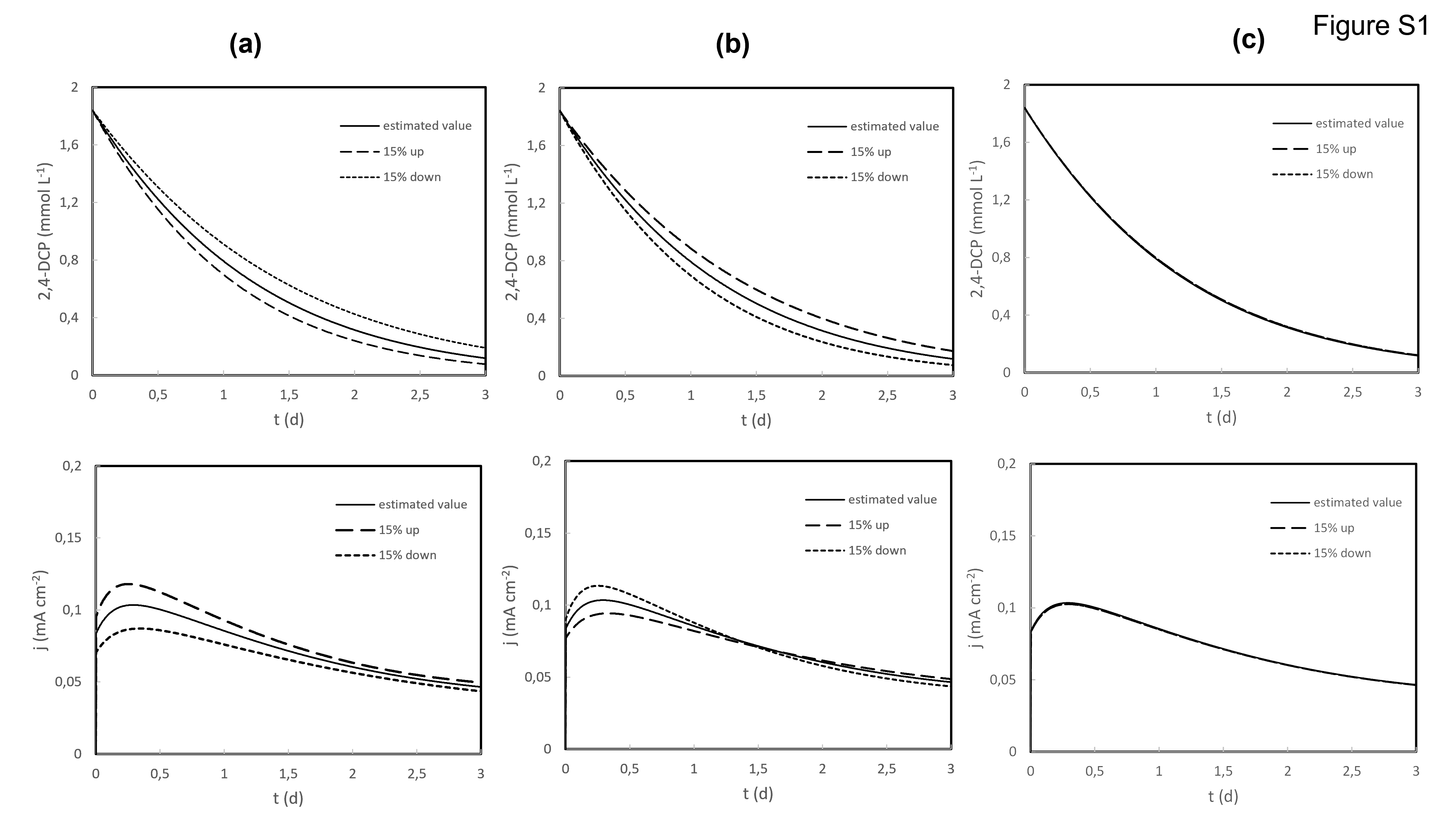

Supplement: Supplementary file 2 — Supplementary file2 (TIF 1002 KB) Simulation results under variations of ±15% in the values of the estimated parameters: (a) changes in µmax,e value; (b) changes in Ks values in electrogenic processes; (c) changes in Ys values in electrogenic processes [file 449_2022_2699_MOESM2_ESM.tif]
